# Magnetic resonance radiomics features and prognosticators in different molecular subtypes of pediatric Medulloblastoma

**DOI:** 10.1371/journal.pone.0255500

**Published:** 2021-07-29

**Authors:** Feng-Chi Chang, Tai-Tong Wong, Kuo-Sheng Wu, Chia-Feng Lu, Ting-Wei Weng, Muh-Lii Liang, Chih-Chun Wu, Wan Yuo Guo, Cheng-Yu Chen, Kevin Li-Chun Hsieh

**Affiliations:** 1 Department of Radiology, School of Medicine, Taipei Veterans General Hospital and National Yang Ming Chiao Tung University, Taipei, Taiwan; 2 Department of Neurosurgery, Taipei Medical University Hospital, Taipei Medical University, Taipei, Taiwan; 3 Department of Neurosurgery, Neurological Institute, School of Medicine, Taipei Veterans General Hospital and National Yang Ming Chiao Tung University, Taipei, Taiwan; 4 Department of Biomedical Imaging and Radiological Science, National Yang Ming Chiao Tung University, Taipei, Taiwan; 5 Department of Medical Imaging, Taipei Medical University Hospital, Taipei, Taiwan; 6 Department of Neurosurgery, Mackay Memorial Hospital, Taipei, Taiwan; 7 Department of Radiology, School of Medicine, College of Medicine, Taipei Medical University, Taipei, Taiwan; University of Pécs Medical School, HUNGARY

## Abstract

**Purpose:**

Medulloblastoma (MB) is a highly malignant pediatric brain tumor. In the latest classification, medulloblastoma is divided into four distinct groups: wingless (WNT), sonic hedgehog (SHH), Group 3, and Group 4. We analyzed the magnetic resonance imaging radiomics features to find the imaging surrogates of the 4 molecular subgroups of MB.

**Material and methods:**

Frozen tissue, imaging data, and clinical data of 38 patients with medulloblastoma were included from Taipei Medical University Hospital and Taipei Veterans General Hospital. Molecular clustering was performed based on the gene expression level of 22 subgroup-specific signature genes. A total 253 magnetic resonance imaging radiomic features were generated from each subject for comparison between different molecular subgroups.

**Results:**

Our cohort consisted of 7 (18.4%) patients with WNT medulloblastoma, 12 (31.6%) with SHH tumor, 8 (21.1%) with Group 3 tumor, and 11 (28.9%) with Group 4 tumor. 8 radiomics gray-level co-occurrence matrix texture (GLCM) features were significantly different between 4 molecular subgroups of MB. In addition, for tumors with higher values in a gray-level run length matrix feature—Short Run Low Gray-Level Emphasis, patients have shorter survival times than patients with low values of this feature (p = 0.04). The receiver operating characteristic analysis revealed optimal performance of the preliminary prediction model based on GLCM features for predicting WNT, Group 3, and Group 4 MB (area under the curve = 0.82, 0.72, and 0.78, respectively).

**Conclusion:**

The preliminary result revealed that 8 contrast-enhanced T1-weighted imaging texture features were significantly different between 4 molecular subgroups of MB. Together with the prediction models, the radiomics features may provide suggestions for stratifying patients with MB into different risk groups.

## Introduction

Medulloblastoma (MB) is the most common primary malignant brain tumor in children and is currently treated on the basis of pathological and clinicoradiological risk stratification [1]. Despite progress in disease management, the overall survival remains dismal with 5 year survival rate around 70~75% [2]. Current therapeutic options have debilitating effects on developing children, highlighting the need for molecularly targeted treatments with reduced toxicity. Recent developments in genome-wide sequencing techniques have improved understanding regarding MB. Many target genes with dysregulated expression and mutation have been identified as possible drivers of MB. Research conducted over the past years has identified four distinct molecular variants, namely wingless (WNT), sonic hedgehog (SHH), Group 3, and Group 4, each with different demographic, and genomic characteristics [3,4]. Improved knowledge of signaling pathways and important oncogenic drivers in these subgroups has elucidated the reasons for suboptimal clinical outcomes and therapeutic resistance. These findings have ushered in a new era of therapeutic optimism, with latest clinical trials now pursued on the basis of the molecular classification of MB. However, no consensus has been reached on which genomic analysis protocol should be applied to identify MB molecular subtypes. Moreover, the heterogeneous spatial distribution of genomic features makes unguided surgical biopsies prone to sampling errors, which may result in misclassification [5,6].

With the development of diagnostic imaging technologies, magnetic resonance imaging (MRI) is now a standard examination procedure employed for pediatric brain tumors. This is because MRI provides a wide range of physiologically meaningful contrasts for distinguishing different components through imaging, thereby improving depiction of heterogeneous patterns of tissue composition within tumors. Several reports have proved that MRI phenotype can be applied to predict the genetic profiling of brain tumors [7]. A study by Perreault et al. indicated that MRI substitute can be a favorable reference for MB molecular subgroups [8]. On the basis of objective radiomics analysis, imaging data can be transformed into a high-dimensional space by using an automatically high-throughput feature extraction algorithm [9]. Quantitative image-based features, including intensity, shape- and size-based, and textural features, can be obtained using this process. Some of these features have been proven to be associated with underlying gene-expression patterns [10].

In this study, multiparametric radiomics MRI analysis was conducted to reveal MB features. Since different MB subgroups have distinct patient demographics, clinical management and disease outcomes [11]. This non-invasive computer-aided quantitative diagnosis procedure may be included as part of the diagnostic workup.

## Materials and methods

### Data collection of patients with MB

This retrospective study was approved by the institutional review boards of Taipei Medical University Hospital and Taipei Veterans General Hospital. The original material and data were collected in compliance with all applicable laws, regulations, and policies for the protection of human participants. Informed consent was obtained from a parent and/or legal guardian for inclusion in the study. The clinical information, frozen tumor specimens, and preoperative magnetic resonance (MR) images of 52 patients with MB were obtained. 14 patients with incomplete clinical data, poor tumor tissue quality, and incomplete or poor-quality MR images were excluded from the research. Only 38 cases were finally included in our study.

### RNA-seq and clustering analysis

For RNA-Seq analysis, total RNA was purified from MB tissues by using the RNeasy Mini Kit (Qiagen). The quality and quantity of RNA were assessed through spectrophotometry and capillary gel electrophoresis, respectively. Next, the stranded mRNA libraries were generated by using the TruSeq Stranded mRNA Library Prep Kit (Illumina). From each sample, 60 ng/μL of poly(A)-selected RNA was run in two lanes of a MiSeq sequencing instrument (Illumina) for 100 cycles of multiplexed paired-end reads. We also performed read pseudoalignment and quantification using Kallisto [12] with 200 bootstraps/paired-end reads aligned against the human genome assembly, GRCh38 transcriptome definition. We extracted the gene-expression table by using the tximport package in RStudio with R, and fragments per kilobase of transcript per million reads data sets were trimmed to obtain the protein coding genes, which was followed by quantile normalization and log2 transformation of each data set [13]. For clustering, we performed unsupervised clustering analysis with the consensus clustering default parameters by using the t-distributed stochastic neighbor embedding (t-SNE) and nonnegative matrix factorization (NMF) [14,15] packages, and validated by 22 subgroup-specific signature genes expression levels (WNT: WIF1, TNC, GAD1, DKK2, and EMX2; SHH: PDLIM3, EYA1, HHIP, ATOH1, and SFRP1; Group 3: IMPG2, GABRA5, NRL, MAB21L2, NPR3, and EYS [EGFL11]; and Group 4: KCNA1, EOMES, KHDRBS2, RBM24, UNC5D, and OAS1) [16]. RNA-Seq results were sent to Taylor’s laboratory in the Hospital for Sick Children, Toronto in helping with counterpart clustering.

### MR image analysis

The MR sequences used for analysis included the axial T1 weighted image (T1WI), axial T2 weighted image (T2WI), axial T2 fluid attenuation inversion recovery (FLAIR) image, contrast-enhanced axial T1WI (CET1), and diffusion weighted image. In total, 14 patients were excluded; 11 lacked preoperative MRI, and 3 had poor MRI quality. Thus, 38 patients were enrolled in our study for imaging analysis. Quantitative imaging feature analysis was performed as follows.

#### Image postprocessing and MR radiomics

**Fig 1:**
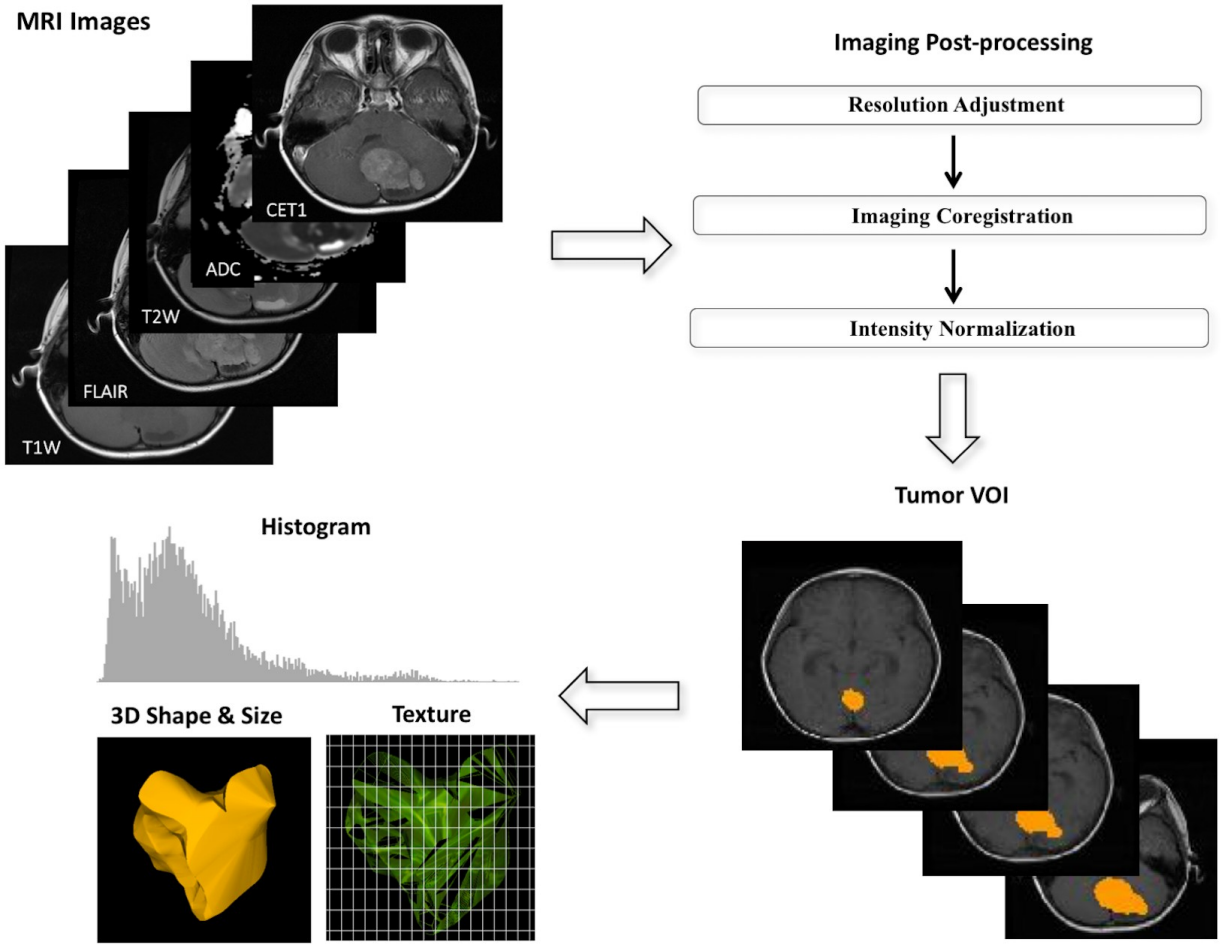
Diagram showing imaging process in radiomics. (a) Multiple postprocessing steps were applied to magnetic resonance images, including image coregistration, adjustment of image resolution, and intensity normalization between patients. (b) The volume of interest covering the total tumor was manually drawn by a neuroradiologist. (c) Three categories of radiomics, namely histogram, three-dimensional geography, and textural analysis, were employed on the processed magnetic resonance images to yield radiomic features.

To prevent variations in textural analytical features resulting from different scanning protocols [17], we applied several postprocessing steps on the MR images to decrease the discrepancy between imaging parameters used in different hospitals. All images were adjusted to voxel size 0.75 × 0.75 × 3.00 mm^3^ without gaps between consecutive slices for each MRI modality. The T2W, T2 FLAIR images, and apparent diffusion coefficient (ADC) maps derived from the diffusion-weighted image were then registered to the CET1s by using a six-parameter rigid body transformation and mutual information algorithm. Image intensity normalization was employed to transform MRI intensity into standardized ranges among all subjects. The volume of interest (gross tumor volume) covering the total tumor volume was delineated by a board-certified neuroradiologist (K.H., with 14 years of experience) who was blinded to the molecular status of all tumors. Pixels included by the defined tumor contour were applied for feature extraction. A diagram illustrating image processing is displayed in Fig 1.

We extracted 57 radiomic image features that describe tumor characteristics based on Aerts’s work [18]. These features can be divided into three categories: tumor intensity (16 features), shape and size (8 features), and texture (33 features). In the first category, tumor intensity information are quantified using first-order statistics, obtained from the histogram of entire tumor voxel intensity values. The second category are features based on the three-dimensional geometry of a tumor, including surface area, volume size, and derived features. The third category consists of three-dimensional texture features that are able to quantify the intratumoral heterogeneity within a full tumor volume. The 16 intensity and 33 textural features were extracted from T1WI, T2WI, T2 FLAIR, CET1, and ADC images to yield 245 features. The eight size and shape features were computed based on the three-dimensional morphology of the tumors. In total, a maximum of 253 MR radiomic features could be generated for each subject. All radiomic features were extracted by using the MR Radiomics Platform (MRP; http://www.ym.edu.tw/~cflu/MRP_MLinglioma.html) which is in line with the regulations of Image Biomarker Standardization Initiative (IBSI) [19]. Detailed formulas of MR radiomic features are provided in Supplementary S1 Table.

#### Handling missing values

After calculation of radiomic features for all subjects, we further performed k-nearest neighbor (k-NN) imputation to compensate for the partially missing values caused by the lack of image sequences for some of the subjects [20]. The k-NN method is a common strategy in which missing values are imputed using values calculated from k nearest neighbors that reach a certain similarity measure. K = 3 was selected in this study since the smallest subgroup (WNT) in our cohort has only 7 cases [21]. The nearest, neighbors are determined based on Euclidean distance function. The superiority of the k-NN method over mean imputation is that the imputed values are influenced by only the most similar cases rather than by all values. After application of the feature extraction protocol in T1WI, T2WI, T2 FLAIR, CET1, ADC map with k-NN imputation, we extracted 253 radiomics features from each tumor.

#### Comparison of radiomics features in 4 molecular groups

The extracted 253 MR radiomic image features were compared to find the difference between the 4 molecular subtypes of medulloblastoma. Dunn’s multiple comparisons test was applied and the significance level (alpha) is set at 0.05.

#### Analyzing the impact of imaging biomarkers on disease survival

We applied univariate and multivariate Cox proportional hazards modeling to investigate the relationships between quantitative radiomic features and overall survival. The Wald test was used to determine the significance of Cox models, and the most significant features were selected to define the radiomic risk subgroups. The log-rank (Mantel–Cox) test was used to compare the survival curves of different subgroups, and hazard ratios were used to report the direction of the survival effect. The significance level (alpha) is set at 0.05.

#### Establishing a prediction model of 4 molecular subtypes of MB

We tried to establish a prediction model to estimate the molecular subtypes of each MB based on different radiomics imaging features. Different feature selection algorithms including minimum redundancy maximum relevance, sequential backward elimination and sequential forward selection were applied to obtain the best future combination [22,23]. A support vector machine (SVM) implemented with nested leave one out cross validation was applied to find the best model [24,25]. The performance of models was scored using several metrics, namely sensitivity, specificity, and accuracy (ACC). However, a meaningful prediction model was hard to be created based on such a small cohort. The detailed methods and preliminary results were presented in supplement S1 Text, S2 and S3 Tables.

## Results

### Clinical characteristics and molecular subtypes of the study cohort

**Fig 2:**
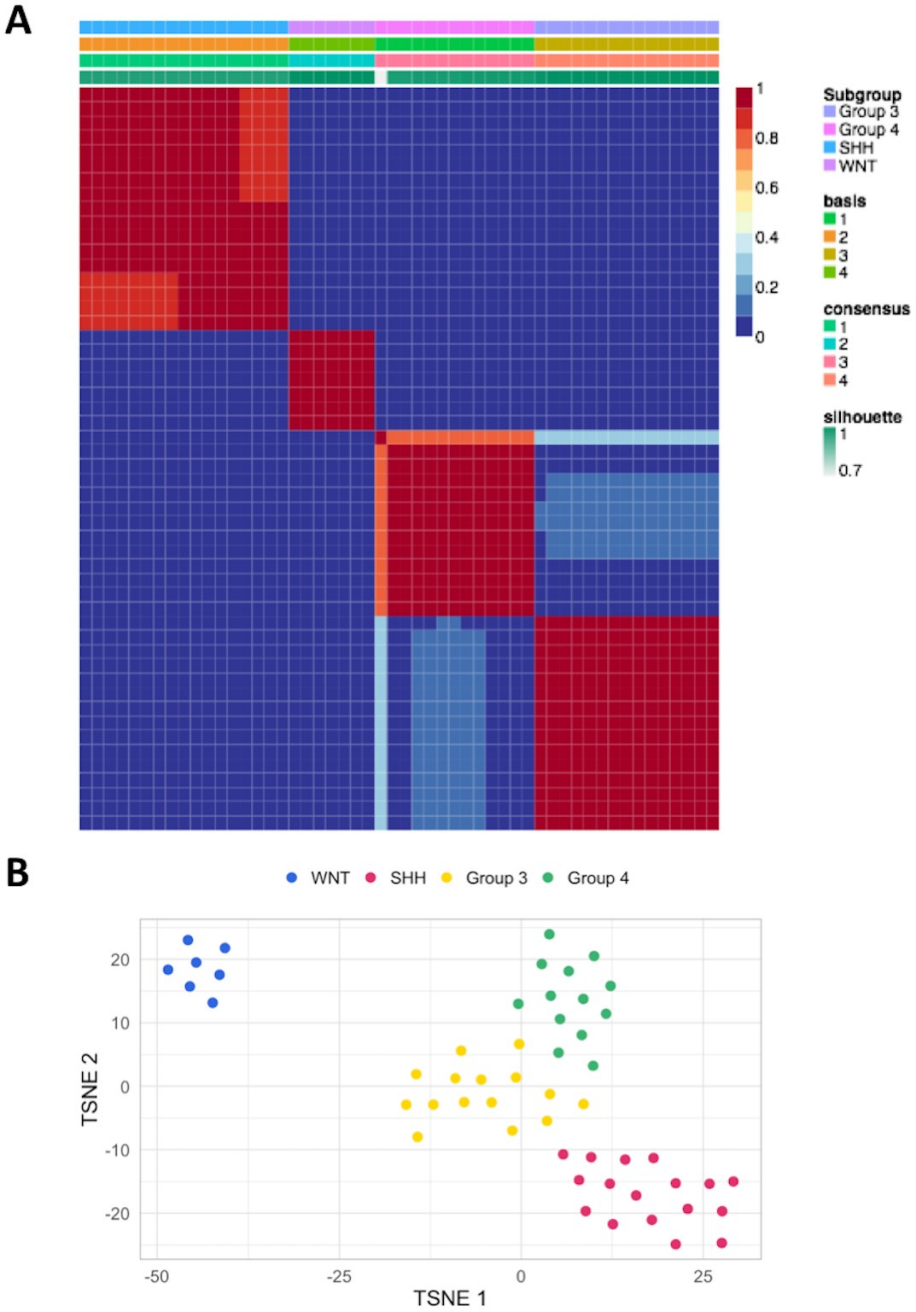
Pediatric MBs were divided into four molecular subgroups. (A) unsupervised NMF and (B) t-SNE analyses of the RNA-Seq gene-expression table. The 52 patients with MB were classified into WNT (7), SHH (17), Group 3 (14), and Group 4 (14). Abbreviations: NMF: Nonnegative matrix factorization; t-SNE: t-distributed stochastic neighbor embedding; WNT: Wingless; SHH: Sonic hedgehog; MB: Medulloblastoma.

In total, 52 children with MBs were collected from the archives. We clustered these cases into 4 molecular subgroups by running unsupervised clustering analysis with NMF and t-SNE according to significantly different expressed genes (Fig 2). Furthermore, we validated the cluster using the 22 subgroup-specific signature gene list [16]. However, 14 patients were excluded because complete preoperative MR images were unavailable. Thus, 38 cases were included in our research, and the mean age of this cohort at diagnosis was 7.8 years. The final subgroups of WNT, SHH, Group 3, and Group 4 consisted of 7 (18.4%), 12 (31.6%), 8 (21.1%), and 11 (28.9%) patients, respectively. Clinical profiles of the included patients are listed in Table 1.

**Table 1:** Clinical profiles of the study cohort.

	WNT	SHH	Group 3	Group 4
Case number	7	12	8	11
Age at diagnosis	6.2 ± 4.1	5.5 ± 4.5	8.3 ± 5.1	10.0 ± 2.9
Boy–girl ratio	1:6	5:7	5:3	6:5
Clinical risk stratification				
Non-met (M0-1) AR	7	7	4	3
Non-met (M0-1) HR	0	3	3	5
Met (M2-3) HR	0	2	1	3
Treatment strategy				
CMT alone	0	1	0	0
RT alone	0	1	1	1
RT + CMT	7	9	7	10

Abbreviations: WNT: Wingless; SHH: Sonic hedgehog; Met: Metastasis; AR: Average risk; HR: High risk; CMT: Chemotherapy; RT: Radiation therapy.

#### Difference of radiomics features in 4 groups

**Fig 3:**
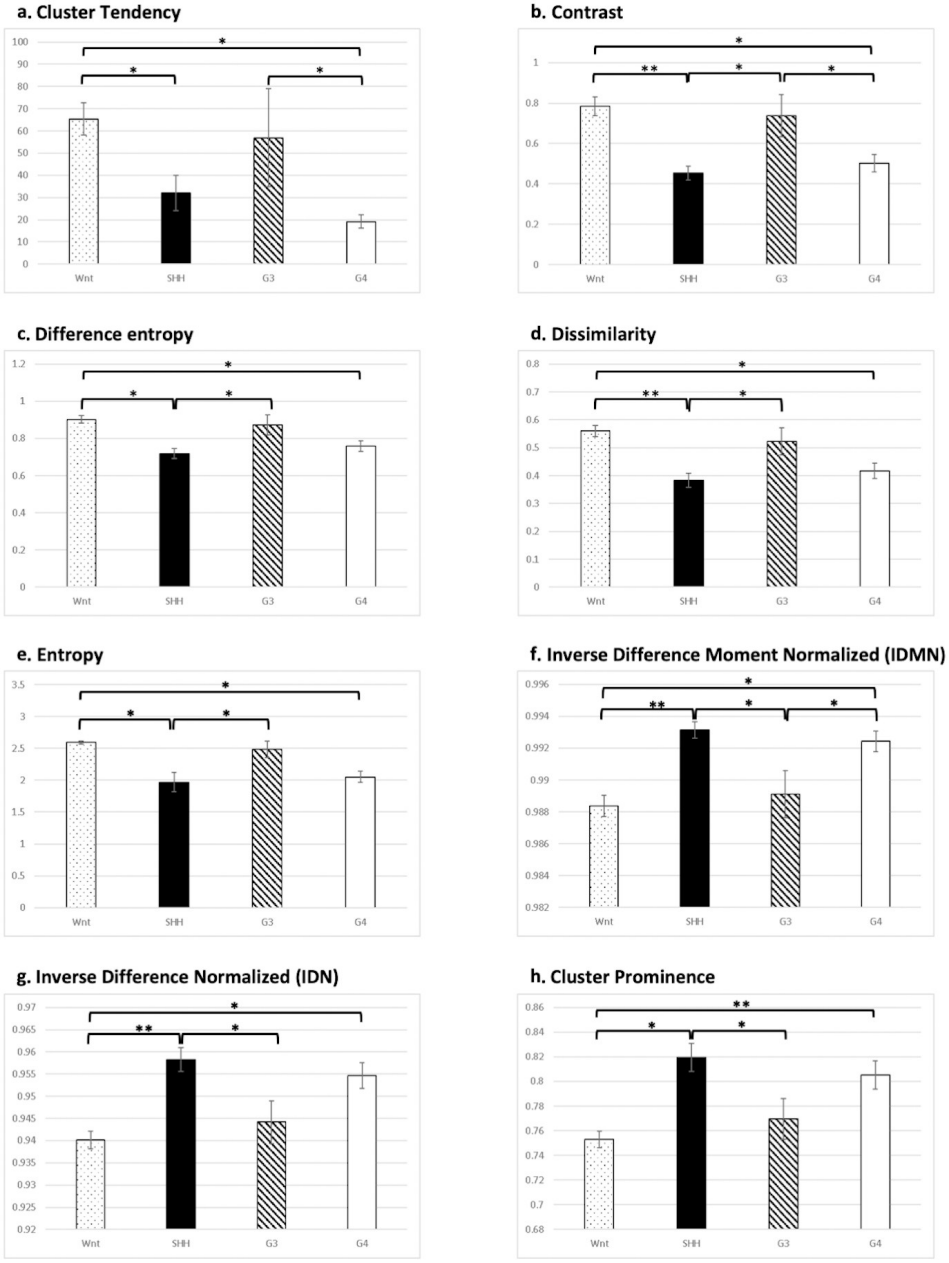
Comparison of the radiomics features which were statistically different between 4 molecular subtypes of medulloblastoma. In first 4 features (A-D) WNT and G3 groups tended to have the higher values than SHH and G4 groups. In the rest 4 features (E-H) SHH and G4 groups tended to have the higher values than WNT and G3 groups. (*: p<0.05; **: p<0.01).

We have compared the 253 radiomic image features between 4 molecular subtypes of medulloblastoma. 6 features were significant different between 3 groups of medulloblastoma and 2 features were significant different between 4 groups of medulloblastoma. In 4 features (Cluster Tendency, Contrast, Difference entropy, Dissimilarity, Fig 3A–3D), WNT and G3 groups tended to have the higher values than SHH and G4 groups. In the rest 4 features (Entropy, Inverse Difference Normalized (IDN), Inverse Difference Moment Normalized (IDMN), Cluster Prominence, Fig 3E–3H) SHH and G4 groups tended to have the higher values than WNT and G3 groups. All of them are textural features illustrating local patterns in tumors.

#### The impact of imaging biomarkers on disease survival

In univariate Cox Proportional-Hazards regression model, 6 radiomic features were proved to be significant correlated to the period of patient survival: Mean (p = 0.04), Median (p = 0.04), Skewness (p = 0.02), Energy (p = 0.01), Short Run Low Gray-Level Emphasis (SRLGLE) (p = 0.01), and Long Run Low Gray-Level Emphasis (LRLGLE) (p = 0.04). However, only Short Run Low Gray-Level Emphasis (SRLGLE) (p = 0.02) were significant correlated to patient overall survival in multivariate Cox Proportional-Hazards regression model.

**Fig 4:**
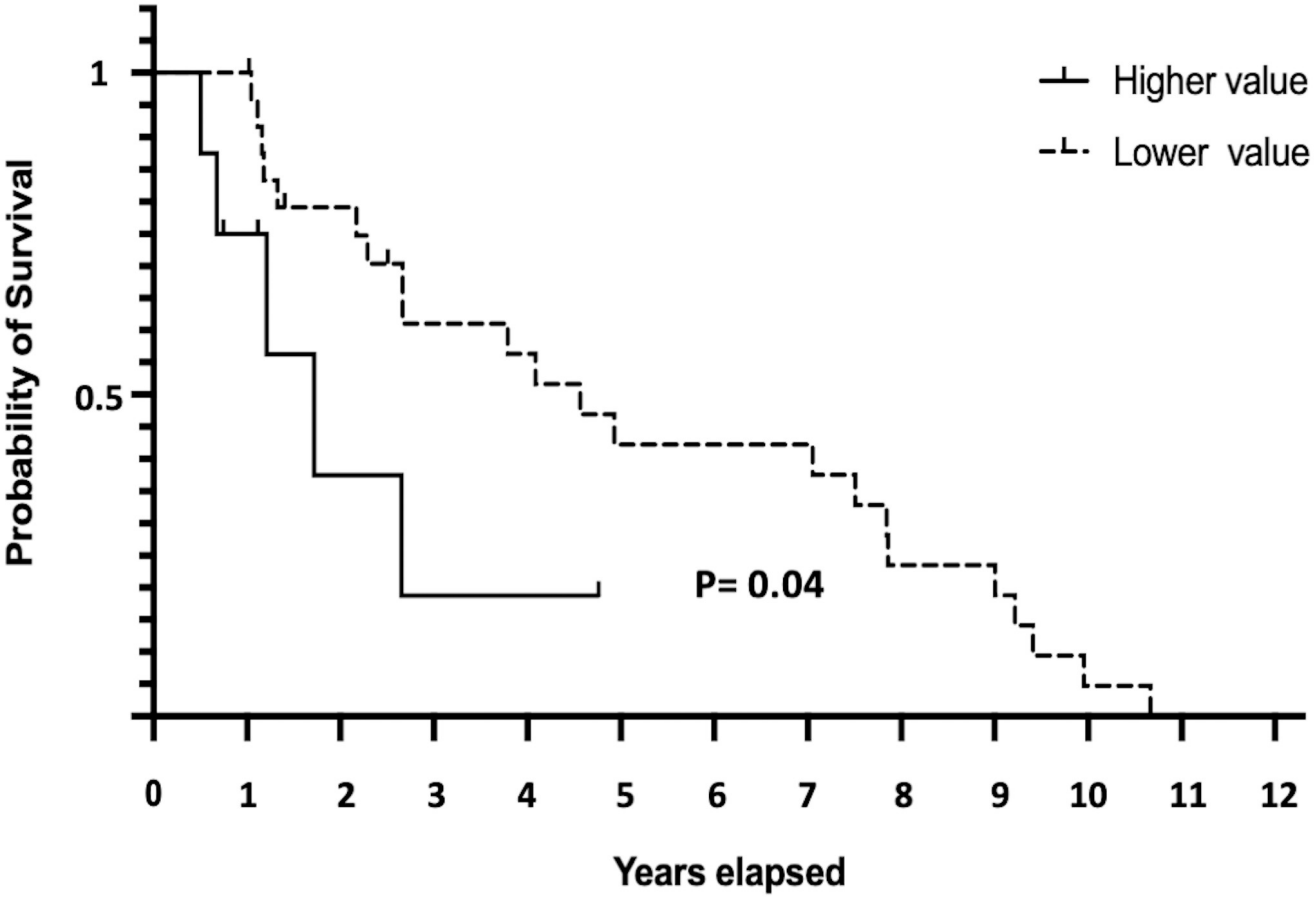
Comparison of survival in MB patients with different radiomics values. For tumors with Energy value > 0.23 or SRLGLE value > 0.08, patients have shorter survival time than tumor don’t have these features (p = 0.04 in Log-rank [Mantel-Cox] test).

SRLGLE was selected as the prognosticator. And we found that in tumors with SRLGLE values higher than the third quartile values 0.08, patients have shorter median survival than tumor don’t have these features (627 vs 1666 days, Hazard ratio: 4.28; 95% Confidence Interval 1.03 to 17.78, p = 0.04, Log-rank [Mantel-Cox] test, Fig 4).

#### Preliminary prediction model for molecular subgroups of MB

**Fig 5:**
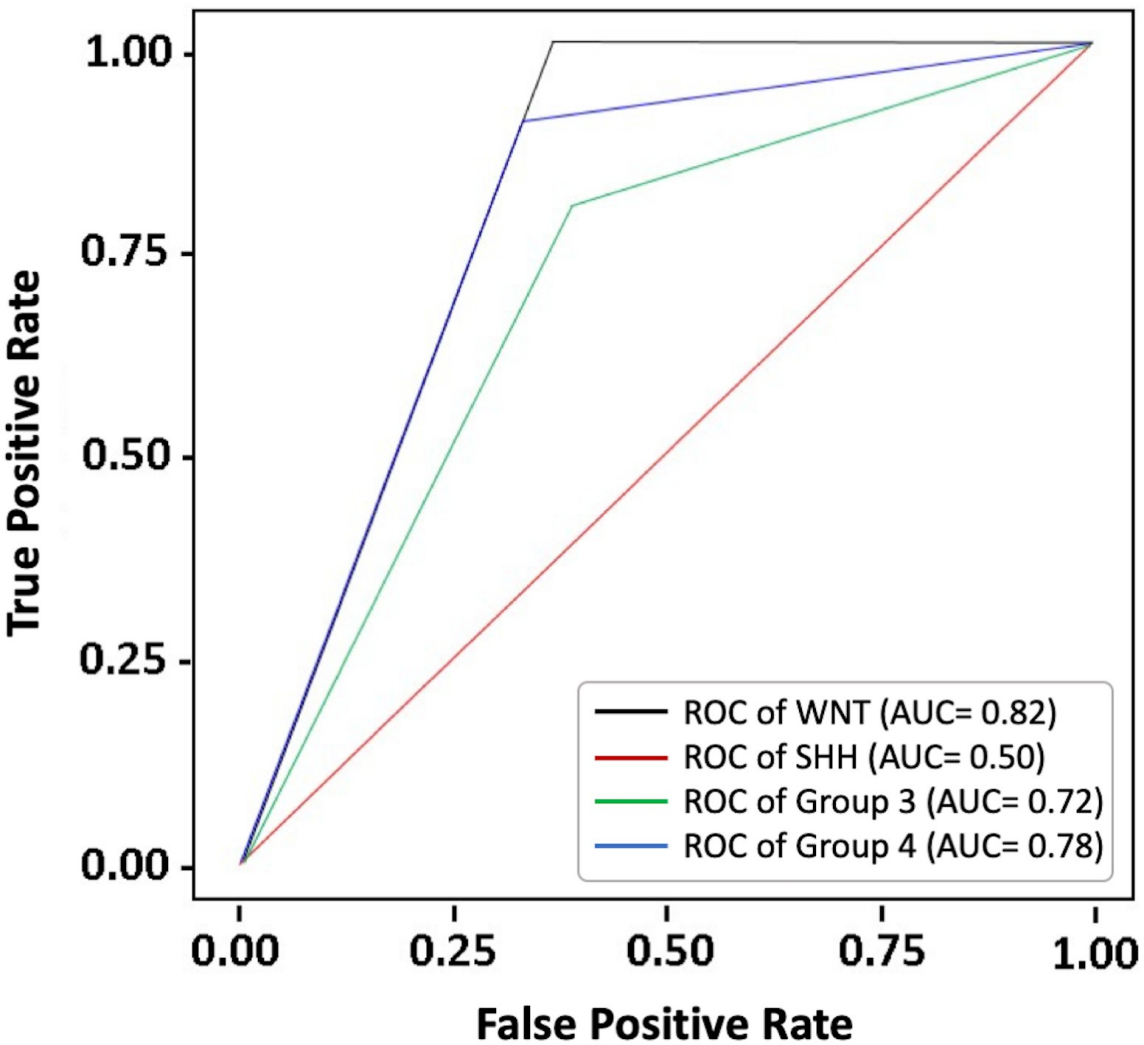
Receiver operating characteristic analysis of the preliminary prediction model. The prediction performance for predicting WNT, SHH, Group 3, and Group 4 MB was illustrated (area under the curve = 0.82, 0.50, 0.72, and 0.78, respectively).

Our result revealed that features extracted from CET1 GLCM features by using sequential forward selection algorithm delivered the highest performance to differentiate 4 molecular subgroups. Receiver operating characteristic analysis revealed optimal performance for predicting WNT, Group 3, and Group 4 MB (area under the curve [AUC] = 0.82, 0.72, and 0.78, respectively). But the prediction performance for SHH MB is suboptimal (AUC = 0.50). (Fig 5) The overall accuracy of the model to estimate the molecular subtypes of each MB is 71%. The model proposed in this study is still in the preliminary stage because a meaningful prediction model was hard to be created based on such a small cohort. The detailed methods and other preliminary results were presented in supplement S1 Text, S2 and S3 Tables.

## Discussion

Our result demonstrated that there is significant difference in MR radiomics features between different MB molecular subtypes. Some features can be applied as prognosticators to predict the outcome of MB.

In 2010, an international panel of experts reached consensus on the four main subgroups of MB [24,26]. This proposal was subsequently adopted in the revised World Health Organization classification of central nervous system tumors published in 2016 [27]. WNT group MBs originate from dorsal brainstem precursors [28] and are featured by activation of the WNT pathway. Mutations in exon 3 of *CTNNB1* and monosomy chromosome 6 are usually detected [29]. SHH group MBs arise from granule cell precursors of the cerebellum and cochlear nucleus [30] and are characterized by activation of the SHH pathway. This group commonly contain mutations in the SHH pathway, including *SMO*, *PTCH* and *SUFU* [31]. Group 3 MBs are featured by recurrent MYC amplifications and almost 20% of patients harboring an MYC amplicon [29]. Overall outcome is poorer compared with the other subgroups. Group 4 is the most common subgroup, and the most common aberration is isochromosome 17q, *MYCN* amplifications, *SNCAIP* duplications and loss of 11q [32]. Molecular subgroups can be identified using various genomic approaches and platforms, including fluorescence *in situ* hybridization or expression array methods, molecular inversion probe single-nucleotide polymorphism assay, or genome-wide methylation [16]. Two independent validated methods performed in accredited laboratories were recommended to reach the correct subgroup assignment [33]. Clinical trials which tailor therapies for each of the subgroups and assess whether this approach can improve outcomes are underway [34].

Linking MRI features with genomic data can help identify not only unique genetic information but also MR phenotypes, which may play a role in genetic dosage alteration [8]. We proposed several quantitative radiomic features in this study for predicting molecular subgroups. These features were categorized according to intensity, morphology, and texture, each providing different perspectives for distinguishing MB molecular subgroups. According to our results, texture features proved the most distinct in differentiating MBs at the molecular level. In previous reports [10,35], researchers likewise observed that texture features in MR images were the most illustrative phenotype for differentiating molecular subtypes of brain glioma. Texture analysis is a defining feature of radiomics that describes the patterns and spatial variations of voxel intensities, calculated from gray-level co-occurrence and gray-level run-length texture matrices, respectively. The classifying features in our cohort were Cluster tendency, Cluster prominence, Entropy, Difference entropy, Dissimilarity, IDN, IDMN, and Contrast. Cluster tendency measures groupings of voxels with similar gray-level values. As a measure of heterogeneity, it places higher weights on neighboring intensity level pairs that deviate more from the mean [18]. Cluster prominence is a measurement of the skewness and asymmetry of gray-level co-occurrence. Higher values imply more asymmetry of the mean value, whereas lower values imply a peak near the mean with less variation [36]. Entropy is a statistical measure of randomness that has been proven to capture intratumoral heterogeneity [37] and has also been associated with tumor staging [38], outcome [39], and expression of molecular pathways. Dissimilarity illustrates local intensity variation, which is defined as the mean absolute difference between the neighboring pairs; a higher value represent greater disparity in intensities among neighboring voxels [40]. IDN (also called similarity) and IDMN (also called homogeneity) are both measures of the local homogeneity [40]. Our study revealed that WNT-MBs have a similar texture value to Group 3 MBs and SHH-MBs have similar texture features to Group 4 MBs. Further studies on the similarities and dissimilarities of these molecular subgroups are warranted.

In our machine learning protocol, features generated from multiparametric MR sequences including CET1, T1W, T2W, T2 FLAIR, and ADC map were input into the algorithms to determine the optimal features for molecular subgroup prediction. The results revealed that the combination of textural features generated from CET1 achieved the highest ACC. We therefore only compared features generated from CET1 images. In CET1 images, several pathological features including tumor necrosis and cysts can be clearly identified. Furthermore, the intensity of contrast enhancement is linked to angiogenesis module activity within the tumor [41,42]. Therefore, it is reasonable that CET1 features may depict the molecular profiles and predict outcome of MB because they provide substantial information regarding tumor characteristics. Iv et al’s report also investigated the prediction model for medulloblastoma [43]. However, their model didn’t take all routine MR sequences into consideration. And even with substantial case number, the prediction accuracy for some molecular subgroup is still suboptimal.

Several radiomic features have been identified as potential prognosticators in our study. Many prognostic factors of medulloblastoma patients has been proposed, including presence of metastases at diagnosis, age < 3 years, extend of resection, residual disease ≥ 1·5 cm^2^, molecular subgroups, craniospinal radiation, and geographic location of therapy [44–47]. Imaging is a routine examination for pediatric brain tumors, with no additional costs associated. By applying radiomic image features, we can quantify all tumor characteristics without risking a biopsy. Therefore, radiomics analysis may be another helpful tool for stratifying patients into different risk groups.

### Limitations

Because the MB occurrence rate is low, this preliminary study was limited by its small sample size. Therefore the accuracy of the established prediction model based on the machine learning is not optimal (S3 Table). Further studies with a larger sample size are warranted. The trained prediction model and developed MRP are available on our website (www.ym.edu.tw/~cflu/MRP_MLinglioma.html), and the complete content will be available after paper publication to encourage researchers worldwide to test these models and refine them accordingly. Another limitation is that we only used images generated from original MR images. Further investigations on other image postprocessing techniques, such as wavelets or ranklets, are necessary.

In conclusion, 8 CET1 texture features were found to be significant different between 4 molecular subgroups of MB. Another CET1 features were found to be a good prognosticator of MB. These features may provide suggestions for further stratifying patients with MB into different risk subgroups.

## Supporting information

S1 Fig Diagram showing nested leave-one-out cross-validation procedure. The data are repeatedly split in testing and decoding sets. The decoding set itself is split in multiple training and validation sets with the same decoding set, forming an inner cross-validation loop used to set the regularization hyperparameter, while the external loop varying the testing set is used to measure the performance of prediction. The process was repeated according to the case number in both inner and external loops. (* 4 cases were excluded during the validation because of their missing values).
(PDF)

S1 Table Employed 57 radiomic features. (PDF)

S2 Table Prediction results obtained in different molecular subgroups using the proposed model based on the sequential forward selection algorithm. The details of the prediction results in different molecular subgroups were demonstrated by using the proposed model based on the selected CET1 features. Overall prediction accuracy was highest in WNT.
(PDF)

S3 Table Accuracy of the prediction model based on different combinations of imaging features extracted from different MR parameters and feature selection algorithms. The Highest accuracy was obtained with sequential forward selection algorithm using CET1 images. (Abbreviations: mRMR, minimum redundancy maximum relevance; SBE, sequential backward elimination; SFS, sequential forward selection).
(PDF)

S1 Text Establishing a prediction model. Taking the RNA-Seq-based molecular subtypes as the gold standard, a prediction model was established to estimate the molecular subtypes of each MB based on the most significant radiomics imaging features defined by the feature selection procedure. The generalizability of the selected features was validated through leave-one-out cross-validation (LOOCV).
(PDF)
