# Supplementary material for: Magnetic resonance radiomics features and prognosticators in different molecular subtypes of pediatric Medulloblastoma
Source: PLoS One. 2021 Jul 29;16(7):e0255500. doi: 10.1371/journal.pone.0255500 (PMC8321137; doi:10.1371/journal.pone.0255500)
Supplement: S1 Fig — The data are repeatedly split in testing and decoding sets. The decoding set itself is split in multiple training and validation sets with the same decoding set, forming an inner cross-validation loop used to set the regularization hyperparameter, while the external loop varying the testing set is used to measure the performance of prediction. The process was repeated according to the case number in both inner and external loops. (* 4 cases were excluded during the validation because of their missing values). (PDF) [file pone.0255500.s001.pdf]

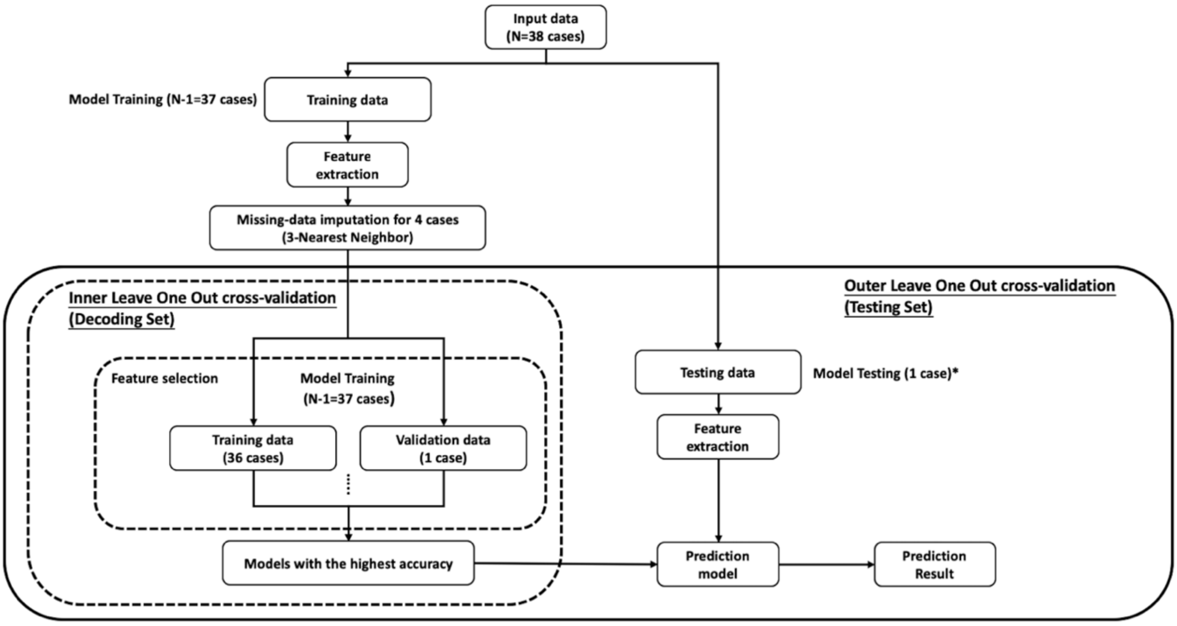

Figure S1. Diagram showing nested leave-one-out cross-validation procedure. The data are repeatedly split in testing and decoding sets. The decoding set itself is split in multiple training and validation sets with the same decoding set, forming an inner cross-validation loop used to set the regularization hyperparameter, while the external loop varying the testing set is used to measure the performance of prediction. The process was repeated according to the case number in both inner and external loops. (* 4 cases were excluded during the validation because of their missing values.)
