# Supplementary material for: Magnetic resonance radiomics features and prognosticators in different molecular subtypes of pediatric Medulloblastoma
Source: PLoS One. 2021 Jul 29;16(7):e0255500. doi: 10.1371/journal.pone.0255500 (PMC8321137; doi:10.1371/journal.pone.0255500)
Supplement: S1 Table — (PDF) [file pone.0255500.s002.pdf]

Table S1. Employed 57 radiomic features.

**Intensity based features (first order statistics)**<br>Let **X** denote the three dimensional image matrix with *N* voxels; $\overline{X}$ be the mean of **X**; **P** be the first order histogram with N_l discrete intensity levels.			
**Feature**	**Formula**	**Feature**	**Formula**
1. Energy	$\sum_{i=1}^{N} \mathbf{X}(i)^2$	2. Entropy	$\sum_{i=1}^{N_l} \mathbf{P}(i) log_2 \mathbf{P}(i)$
3. Kurtosis	$\frac{\frac{1}{N}\sum_{i=1}^{N}(\mathbf{X}(i)-\bar{X})^4}{\left(\sqrt{\frac{1}{N}\sum_{i=1}^{N}(\mathbf{X}(i)-\bar{X})^2}\right)^2}$	4. Maximum	$\max(\mathbf{X})$
5. Mean	$\frac{1}{N}\sum_{i=1}^{N} \mathbf{X}(i)$	6. Mean absolute deviation	$\frac{1}{N}\sum_{i=1}^{N} \mathrm{abs}(\mathbf{X}(i)-\bar{X})$
7. Median	$\mathrm{median}(\mathbf{X})$	8. First quartile	Value that splits off the lowest 25% of data from the highest 75%
9. Third quatile	Value that splits off the highest 25% of data from the highest 75%	10. Minimum	$\min(\mathbf{X})$
11. Range	$\max(\mathbf{X}) - \min(\mathbf{X})$	12. Root mean square (RMS)	$\sqrt{\frac{\sum_{i=1}^{N} \mathbf{X}(i)^2}{N}}$
13. Skewness	$\frac{\frac{1}{N}\sum_{i=1}^{N}(\mathbf{X}(i)-\bar{X})^3}{\left(\sqrt{\frac{1}{N}\sum_{i=1}^{N}(\mathbf{X}(i)-\bar{X})^2}\right)^3}$	14. Standard deviation	$\sqrt{\frac{1}{N-1}\sum_{i=1}^{N}(\mathbf{X}(i)-\bar{X})^2}$
15. Uniformity	$\sum_{i=1}^{N_l} \mathbf{P}(i)^2$	16. Variance	$\frac{1}{N-1}\sum_{i=1}^{N}(\mathbf{X}(i)-\bar{X})^2$
**Shape/Size based features**<br>Let *V* denote the tumor volume and *A* the surface area of the volume.			
17. Compactness 1	$\frac{V}{\sqrt{\pi}A^{2/3}}$	18. Compactness 2	$36\pi\frac{V^2}{A^3}$
19. Maximum 3D diameter	The largest pairwise Euclidean distance between voxels on the surface of the tumor volume.	20. Spherical disproportion	$\frac{A}{4\pi R^2}$
21. Sphericity	$\frac{\pi^{\frac{1}{3}}(6V)^{\frac{2}{3}}}{A}$	22. Surface area	$A = \sum_{i=1}^{Ns}\frac{1}{2}\lvert a_i b_i \times a_i c_i \rvert$<br>*Ns* is the total number

			of triangles covering the surface and a, b and c are vertices of the triangles.
23. Surface to volume ratio	$\frac{A}{V}$	24. Volume	The number of pixels in the tumor region multiplied by the voxel size.

Table S1 (cont.). Employed 57 radiomic features.

**Textural features (gray-level co-occurrence matrix based features)** Let $\mathbf{P}(\delta,\alpha)$ be the co-occurrence matrix for an arbitrary distance δ and direction α; N_g be the number of discrete intensity levels in the image; $p_x(i)$ be the marginal row probabilities; $p_y(i)$ be the marginal column probabilities; μ_x be the mean of p_x; μ_y be the mean of p_y; σ_x be the standard deviation of p_x; σ_y be the standard deviation of p_y; HXY be the entropy of $\mathbf{P}$; HX be the entropy of p_x; HY be the entropy of p_y; $p_{x+y}(k)=\sum_{i=1}^{N_g}\sum_{j=1}^{N_g}\mathbf{P}(i,j), i+j=k, k=2,3,\ldots,2N_g$; $p_{x-y}(k)=\sum_{i=1}^{N_g}\sum_{j=1}^{N_g}\mathbf{P}(i,j), \lvert i-j\rvert=k, k=0,1,\ldots,N_g-1$; $\mathrm{HX}=-\sum_{i=1}^{N_g}p_x(i)\log(p_x(i))$, $\mathrm{HY}=-\sum_{i=1}^{N_g}p_y(i)\log\left(p_y(i)\right)$; $\mathrm{HXY1}=-\sum_{i=1}^{N_g}\sum_{j=1}^{N_g}\mathbf{P}(i,j)\log\left(p_x(i)p_y(j)\right)$, $\mathrm{HXY2}=-\sum_{i=1}^{N_g}\sum_{j=1}^{N_g}p_x(i)p_y(j)\log\left(p_x(i)p_y(j)\right)$			
25. Autocorrelation	$\sum_{i=1}^{N_g}\sum_{j=1}^{N_g}ij\mathbf{P}(i,j)$	26. Cluster Prominence	$\sum_{i=1}^{N_g}\sum_{j=1}^{N_g}[\boldsymbol{i}+\boldsymbol{j}-\boldsymbol{\mu_x}-\boldsymbol{\mu_y}]^4\mathbf{P}(i,j)$
27. Cluster Shade	$\sum_{i=1}^{N_g}\sum_{j=1}^{N_g}[\boldsymbol{i}+\boldsymbol{j}-\boldsymbol{\mu_x}-\boldsymbol{\mu_y}]^3\mathbf{P}(i,j)$	28. Cluster Tendency	$\sum_{i=1}^{N_g}\sum_{j=1}^{N_g}[\boldsymbol{i}+\boldsymbol{j}-\boldsymbol{\mu_x}-\boldsymbol{\mu_y}]^2\mathbf{P}(i,j)$
29. Contrast	$\sum_{i=1}^{N_g}\sum_{j=1}^{N_g}\lvert\boldsymbol{i}-\boldsymbol{j}\rvert^2\mathbf{P}(i,j)$	30. Correlation	$\sum_{i=1}^{N_g}\sum_{j=1}^{N_g}\frac{ij\mathbf{P}(i,j)-\mu_x(i)\mu_y(j)}{\sigma_x(i)\sigma_y(j)}$
31. Difference entropy	$\sum_{i=0}^{N_g-1}p_{x-y}(i)log_2[p_{x-y}(i)]$	32. Dissimilarity	$\sum_{i=1}^{N_g}\sum_{j=1}^{N_g}\lvert\boldsymbol{i}-\boldsymbol{j}\rvert\mathbf{P}(i,j)$
33. Energy	$\sum_{i=1}^{N_g}\sum_{j=1}^{N_g}[\mathbf{P}(i,j)]^2$	34. Entropy (HXY)	$-\sum_{i=1}^{N_g}\sum_{j=1}^{N_g}\mathbf{P}(i,j)\log_2(\mathbf{P}(i,j))$
35. Homogeneity 1	$\sum_{i=1}^{N_g}\sum_{j=1}^{N_g}\frac{\mathbf{P}(i,j)}{1+\lvert i-j\rvert}$	36. Homogeneity 2	$\sum_{i=1}^{N_g}\sum_{j=1}^{N_g}\frac{\mathbf{P}(i,j)}{1+\lvert i-j\rvert^2}$
37. Informational measure of correlation 1	$\frac{HXY-HXY1}{\max(HX,HY)}$	38. Informational measure of correlation 2	$\sqrt{1-e^{-2(HXY2-HXY)}}$
39. Inverse Difference Moment Normalized	$\sum_{i=1}^{N_g}\sum_{j=1}^{N_g}\frac{\mathbf{P}(i,j)}{1+\left(\frac{\lvert i-j\rvert^2}{N^2}\right)}$	40. Inverse Difference Normalized	$\sum_{i=1}^{N_g}\sum_{j=1}^{N_g}\frac{\mathbf{P}(i,j)}{1+\left(\frac{\lvert i-j\rvert}{N}\right)}$

41. Inverse variance	$\sum_{i=1}^{N_g}\sum_{j=1}^{N_g}\frac{\mathbf{P}(i,j)}{\lvert i-j\rvert^2}, i \neq j$	42. Maximum Probability	$\max(\mathbf{P}(i,j))$
43. Sum average	$\sum_{i=2}^{2N_g}[i\mathbf{P}_{x+y}(i)]$	44. Sum entropy	$-\sum_{i=2}^{2N_g}\mathbf{P}_{x+y}(i)log_2[\mathbf{P}_{x+y}(i)]$
45. Sum variance	$\sum_{i=2}^{2N_g}(i-SE)^2\mathbf{P}_{x+y}(i)$	46. Variance	$\sum_{i=1}^{N_g}\sum_{j=1}^{N_g}(i-\mu)^2\mathbf{P}(\mathbf{i},\mathbf{j})$
**Textural features (gray-level run-length matrix based features)** Let $p(i,j\vert\theta)$ be the (i,j)th entry in the given run-length matrix p for a direction θ; N_g be the number of discrete intensity levels in the image; N_r be the number of different run lengths.			
47. Short Run Emphasis	$\frac{\sum_{i=1}^{N_g}\sum_{j=1}^{N_r}\left[\frac{p(i,j\vert\theta)}{j^2}\right]}{\sum_{i=1}^{N_g}\sum_{j=1}^{N_r}p(i,j\vert\theta)}$	48. Long Run Emphasis	$\frac{\sum_{i=1}^{N_g}\sum_{j=1}^{N_r}j^2p(i,j\vert\theta)}{\sum_{i=1}^{N_g}\sum_{j=1}^{N_r}p(i,j\vert\theta)}$
49. Gray Level Non-Uniformity	$\frac{\sum_{i=1}^{N_g}\left[\sum_{j=1}^{N_r}p(i,j\vert\theta)\right]^2}{\sum_{i=1}^{N_g}\sum_{j=1}^{N_r}p(i,j\vert\theta)}$	50. Run Length Non-Uniformity	$\frac{\sum_{j=1}^{N_r}\left[\sum_{i=1}^{N_g}p(i,j\vert\theta)\right]^2}{\sum_{i=1}^{N_g}\sum_{j=1}^{N_r}p(i,j\vert\theta)}$
51. Run Percentage	$\sum_{i=1}^{N_g}\sum_{j=1}^{N_r}\frac{p(i,j\vert\theta)}{N_p}$	52. Low Gray Level Run Emphasis	$\frac{\sum_{i=1}^{N_g}\sum_{j=1}^{N_r}\left[\frac{p(i,j\vert\theta)}{i^2}\right]}{\sum_{i=1}^{N_g}\sum_{j=1}^{N_r}p(i,j\vert\theta)}$
53. High Gray Level Run Emphasis	$\frac{\sum_{i=1}^{N_g}\sum_{j=1}^{N_r}i^2p(i,j\vert\theta)}{\sum_{i=1}^{N_g}\sum_{j=1}^{N_r}p(i,j\vert\theta)}$	54. Short Run Low Gray Level Emphasis	$\frac{\sum_{i=1}^{N_g}\sum_{j=1}^{N_r}\left[\frac{p(i,j\vert\theta)}{i^2j^2}\right]}{\sum_{i=1}^{N_g}\sum_{j=1}^{N_r}p(i,j\vert\theta)}$
55. Short Run High Gray Level Emphasis	$\frac{\sum_{i=1}^{N_g}\sum_{j=1}^{N_r}\left[\frac{p(i,j\vert\theta)i^2}{j^2}\right]}{\sum_{i=1}^{N_g}\sum_{j=1}^{N_r}p(i,j\vert\theta)}$	56. Long Run Low Gray Level Emphasis	$\frac{\sum_{i=1}^{N_g}\sum_{j=1}^{N_r}\left[\frac{p(i,j\vert\theta)j^2}{i^2}\right]}{\sum_{i=1}^{N_g}\sum_{j=1}^{N_r}p(i,j\vert\theta)}$
57. Long Run High Gray Level Emphasis	$\frac{\sum_{i=1}^{N_g}\sum_{j=1}^{N_r}i^2j^2p(i,j\vert\theta)}{\sum_{i=1}^{N_g}\sum_{j=1}^{N_r}p(i,j\vert\theta)}$		
