# Supplementary material for: Magnetic resonance radiomics features and prognosticators in different molecular subtypes of pediatric Medulloblastoma
Source: PLoS One. 2021 Jul 29;16(7):e0255500. doi: 10.1371/journal.pone.0255500 (PMC8321137; doi:10.1371/journal.pone.0255500)
Supplement: S2 Table — The details of the prediction results in different molecular subgroups were demonstrated by using the proposed model based on the selected CET1 features. Overall prediction accuracy was highest in WNT. (PDF) [file pone.0255500.s003.pdf]

Table S2: Prediction Results Obtained in Different Molecular Subgroups Using the Proposed Model Based on the Sequential Forward Selection Algorithm

	**WNT**	**SHH**	**Group 3**	**Group 4**
**Sensitivity (%)**	71	75	38	90
**Specificity (%)**	94	85	90	93
**Accuracy (%)**	90	82	79	92

The details of the prediction results in different molecular subgroups were demonstrated by using the proposed model based on the selected CET1 features. Overall prediction accuracy was highest in WNT.

Abbreviations: WNT: wingless; SHH: sonic hedgehog.
