# Supplementary material for: Magnetic resonance radiomics features and prognosticators in different molecular subtypes of pediatric Medulloblastoma
Source: PLoS One. 2021 Jul 29;16(7):e0255500. doi: 10.1371/journal.pone.0255500 (PMC8321137; doi:10.1371/journal.pone.0255500)
Supplement: S3 Table — The Highest accuracy was obtained with sequential forward selection algorithm using CET1 images. (Abbreviations: mRMR, minimum redundancy maximum relevance; SBE, sequential backward elimination; SFS, sequential forward selection). (PDF) [file pone.0255500.s004.pdf]

Table S3: Accuracy of the Prediction Model Based on Different Combinations of Imaging Features Extracted from Different MR Parameters and Feature Selection Algorithms

	All features	mRMR	SBE	SFS
**CET1**	59%	65%	62%	71%
**T1W**	51%	66%	57%	66%
**T2W**	50%	58%	55%	63%
**ADC**	56%	61%	64%	67%
**FLAIR**	41%	47%	47%	50%
**CET1+T1W**	58%	61%	58%	68%
**CET1+T2W**	53%	59%	65%	68%
**CET1+FLAIR**	50%	53%	57%	63%
**CET1+ADC**	63%	63%	66%	69%
**CET1+T1W+T2W**	52%	55%	55%	58%
**CET1+T1W+T2W+ADC**	47%	50%	50%	53%
**CET1+T1W+T2W+ADC+FLAIR**	46%	50%	54%	54%
**Mean accuracy**	52.2%	57.3%	57.5%	62.5%

The Highest accuracy was obtained with sequential forward selection algorithm using CET1 images. (Abbreviations: mRMR, minimum redundancy maximum relevance; SBE, sequential backward elimination; SFS, sequential forward selection.)
