# Supplementary material for: Magnetic resonance radiomics features and prognosticators in different molecular subtypes of pediatric Medulloblastoma
Source: PLoS One. 2021 Jul 29;16(7):e0255500. doi: 10.1371/journal.pone.0255500 (PMC8321137; doi:10.1371/journal.pone.0255500)
Supplement: S1 Text — Taking the RNA-Seq-based molecular subtypes as the gold standard, a prediction model was established to estimate the molecular subtypes of each MB based on the most significant radiomics imaging features defined by the feature selection procedure. The generalizability of the selected features was validated through leave-one-out cross-validation (LOOCV). (PDF) [file pone.0255500.s005.pdf]

Supplement Materials

S1 Text. Establishing a Prediction Model

Taking the RNA-Seq-based molecular subtypes as the gold standard, a prediction model was established to estimate the molecular subtypes of each MB based on the most significant radiomics imaging features defined by the feature selection procedure. The generalizability of the selected features was validated through leave-one-out cross-validation (LOOCV).

Feature selection

Different methods of feature selection were employed to determine the best combination of imaging features in our prediction model. Feature selection stopped upon achieving the smallest error rate. The generalizability of the selected features was validated through leave-one-out cross-validation (LOOCV).

- **Minimum redundancy maximum relevance feature selection**

Minimum redundancy maximum relevance (mRMR) was first applied for feature selection; mRMR tends to select features with high correlation with the label and low correlation between themselves (20). For continuous features, the F-statistic was used to calculate the correlation with the label. Thereafter, features were selected one by one using greedy search to maximize the objective function, which is a function of relevance and redundancy. Selecting a subset of features based on their importance and similarity can formulate an optimization problem. To evaluate the importance of each feature, we used the mean average precision of each feature as a ranking model for training data and Pearson correlation coefficient between the feature and class label.

- **Sequential backward elimination**

Backward elimination is the simplest method in feature selection (21). The algorithm starts from the complete set of all available features and then removes features one at a time, beginning with the feature with the highest p-value (provided the p-value exceeds a given threshold, usually 5%). The model is refit after each elimination and process loops until a model is identified in which each feature's p-value falls below the threshold.

- **Sequential forward selection**

Sequential forward selection is one of the commonly used heuristic methods for feature selection [1]. In the present study, it involved several steps: First, a k-NN classifier was used to test the data, and LOOCV was employed for recognition rate estimate. Second, the first feature to have the highest LOOCV recognition rate among all features was selected. Third, among all unselected features, the feature with the highest recognition rate together with the selected features was selected. The third step was repeated until the recognition result achieved the smallest error rate.

- **Support vector machine**

A support vector machine (SVM) is a supervised machine learning technique that identifies a decision boundary (separating hyperplane) for either classification or regression [2]. The decision boundary in the SVM classification is calculated by maximizing the margin between the separating hyperplane and support vectors, a subset of training samples closed to the hyperplane. When dealing with a nonlinear condition, the sample space can be mapped to a higher dimensional feature space, where the linear separation can be applied for the classification by using appropriate kernel functions.

- **Nested leave-one-out cross-validation**

Since we have a finite amount of cases, we applied a nested leave-one-out cross-validation procedure [3]: the data are repeatedly split in a testing set and a decoding set to perform decoding. The decoding set itself is split in multiple training and validation sets with the same decoding set, forming an inner "nested" cross-validation loop used to set the regularization hyperparameter, while the external loop varying the testing set is used to measure the performance of prediction (Fig. ~~2~~ S1). For a data set of n patients, the process was repeated n times in both inner and external loops. The performance of models was scored using several metrics, namely sensitivity (SN), specificity (SP), and accuracy (ACC). The scores of SN, SP, and ACC were calculated at the cutoff point when half the subclassifiers returned a positive decision.

Prediction result based on machine-learning algorithm

Our result revealed that features extracted from CET1 by using sequential forward selection algorithm delivered the highest performance with an overall ACC of 71% to differentiate 4 molecular subgroups. The final features applied in the prediction model were cluster prominence, cluster tendency and low gray-level run emphasis (LGLRE). All of the features

selected from our machine learning protocol are textural features. All the morphological and intensity features were not included in any of our prediction models. Table S2 and S3 shows individual prediction results in the individual molecular subtypes of MB for various combinations of MR sequences and feature selection algorithms.
